# A novel pupilloplasty in crescent-shaped suturing pattern for coloboma and traumatic iris defects

**DOI:** 10.1186/s12886-023-02853-0

**Published:** 2023-03-24

**Authors:** Xiaoming Yao, Qian Kang, Wei Qi, Yuan Liu, Xiaoping Zhou, Mingwu Wang, Yukun Yang, Fengjiao Zhu, Wenchao Cao

**Affiliations:** 1 Department of Ocular Surface Disorders and Cornea, Chengdu Aidi Eye Hospital, Sichuan, 610000 China; 2 Shenzhen Huaxia Eye Hospital, Guangdong, China; 3 Guizhou Jinglang Eye Hospital, Guiyang, Guizhou, China; 4 Shenzhen Eye Bank, Shenzhen, Guangdong China; 5 Department of Ophthalmology and Vision Science, University of Arizona, Tucson, AZ USA; 6 Shanghai Pudong New Area Eye and Dental Disease Prevention & Treatment Center, Shanghai, 201399 China; 7 Shenzhen Eye Hospital, Shenzhen, 518000 Guangdong China

**Keywords:** Iris defect, Coloboma, Pupilloplasty, Crescent

## Abstract

**Objective:**

**Purpose:**

To observe the safety and effect of the C-pupilloplasty for the treatment of iris coloboma and traumatic iris defects.

**Methods:**

A total of 21 cases (21 eyes) with iris coloboma or traumatic iris defects who underwent C-pupilloplasty (a single-pass three-throw technique) from Feb. 2016 to Mar. 2020 were analyzed retrospectively. Uncorrected visual acuity, refraction, corneal topographic keratometry and endothelial cell density were examined.

**Results:**

All the patients were successfully treated, and a central and round pupil was restored. The mean follow-up duration was 8.76 ± 3.58 months (ranging from 2 to 14 months). All patients had round or round-like pupils with a diameter less than or equal to 3 mm after the C-pupilloplasty. Very slightly endothelial loss, negligible symptoms such as glare, distortion, dizziness and photophobia were observed.

**Conclusion:**

We introduced a new technique of pupilloplasty (C-pupilloplasty) which could be a more straight forward and more effective treatment for iris coloboma and traumatic iris defect.

## Introduction

Iris and pupillary defects cause unwanted optical problems, such as aberrations, monocular diplopia, glare, halos or photophobia, etc. [1, 2], which also occur in conditions such as glaucoma, intraocular tumour, iris atrophy, ocular trauma, iridodialysis, congenital iris coloboma and posterior iris adhesion. In addition, a cosmetic discrepancy between both eyes can lead to psychophysiological problems in these patients [3, 4]. When cataract or intraocular lens surgery is performed in patients with traumatic pupil dilation and iridodialysis, the optic edges of the implanted lens are often exposed, regardless if the lens is within the bag or fixed by sutures, causing the patients to feel intolerable halo or inability to focus on objects after surgery.

There are two main treatments for iris coloboma. One is iris suturing, and sectoral iris defect less than two clock hours without hyperplasia, rubeosis and fibrosis is amendable to repair by this method [5–8]. But if the iris defect size is larger than two clock hours, or when iris atrophy, iridocorneal endothelial syndrome and congenital aniridia are present, artificial iris implantation should be considered [9–12].

Previous studies had reported satisfactory outcomes with several conventional approaches which make the deformed pupil small, round and more centered [5–8]. We here describe a novel technique of iris suturing which provides an easy and effective alternative treatment for iris coloboma or traumatic iris defects. In our study, a total of 21 cases with iris coloboma or traumatic iris defects underwent the close-ended double thread suturing technique and all the patients were successfully treated. Because the threads run like a crescent moon, we call this new technique as C-pupilloplasty.

## Methods

### Study design

We retrospectively reviewed the medical record of patients with iris coloboma or traumatic iris defects who underwent C-pupilloplasty at our eye hospital from Feb. 2016 to Aug. 2019. Only iris coloboma or traumatic iris defect was less than ≤ 10° or 2 clock hours were included. Patients with uveitis, oversized iris defect, atrophic iris or patients who were not willing to participate in the study were excluded. A Total of 21 patients (21 eyes) were enrolled in this study. The study adhered to the tenets of the Declaration of Helsinki and the ethics protocol was approved by our hospital. Written informed consent from the patients was obtained.

**Fig. 1:**
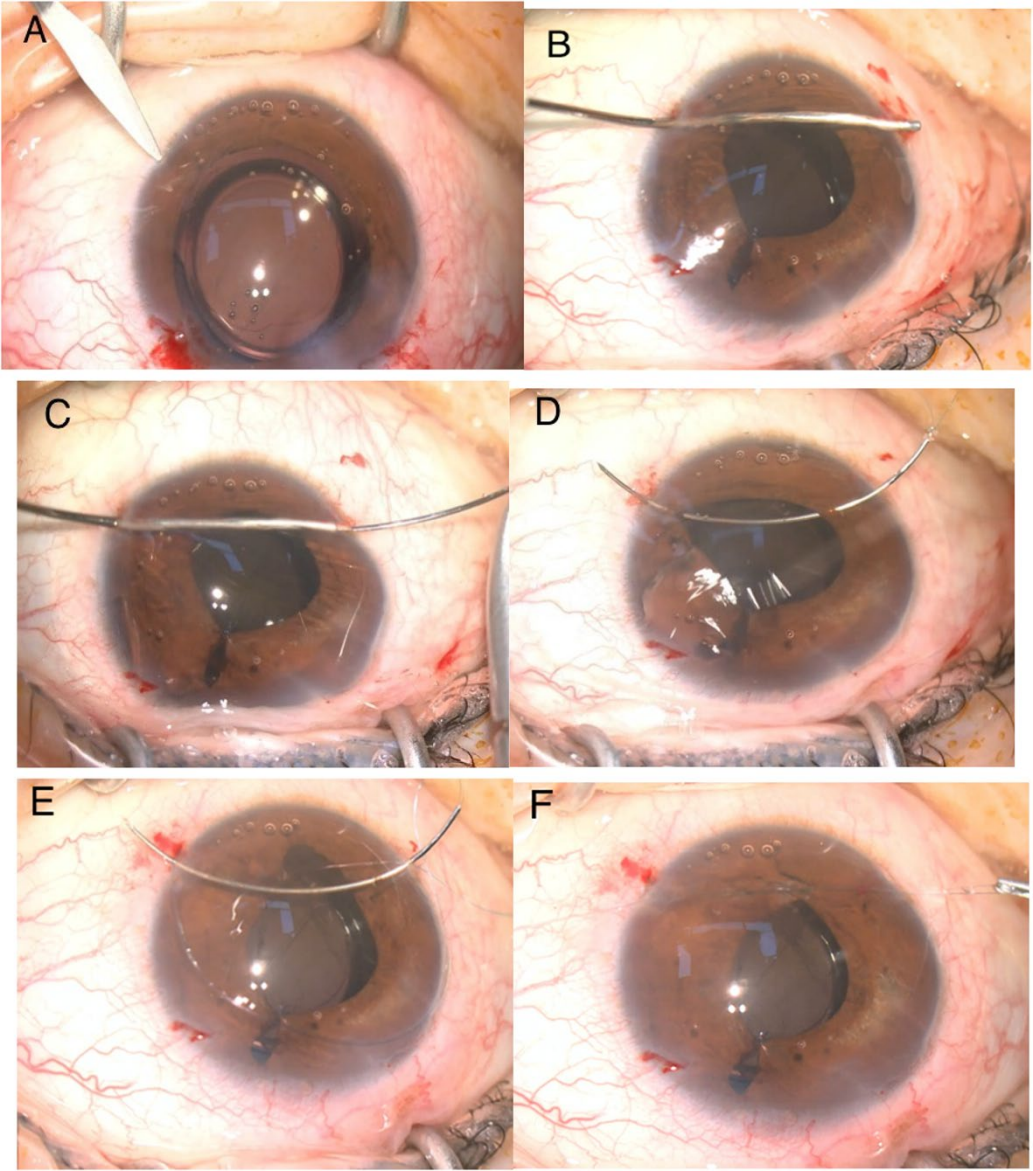
**A**. Two paracentesis are made on both corneal limbus perpendicular to iris defect. **B**. A 27-gauge blunt bend cannula is introduced from distal centesis to proximal one. **C**. The tip docked into the lumen of cannula and moved into anterior chamber. **D**. The tip unreeved from the barrel when it goes into the anterior chamber again, once passes through the iris, tip pulled out from the distal paracentesis but tail remains in anterior chamber. **E**. Wraps the thread for two throw around the suture near the tail outside the proximalcentesis, slides the loops on iris. **F**. It pulls two ends of suture and tie knot in the opposite directions equally and synchronously and cut the suture 0.5 mm away from the knot

**Fig. 2:**
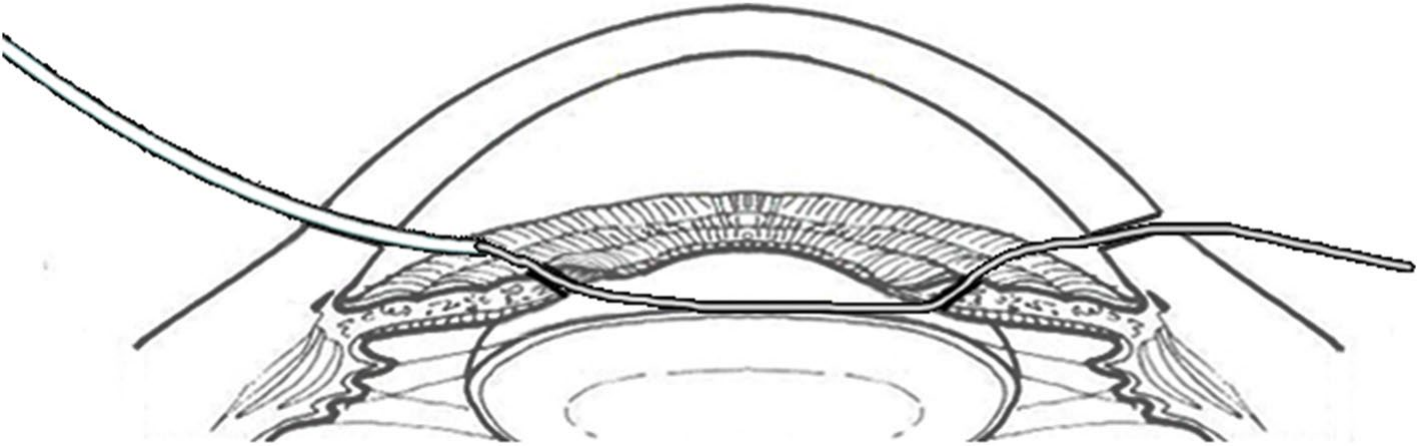
Under the guidance and assistance of cannula, the needle enters the anteriorchamber from the proximal paracentesis, passes through both sides of the defected iris,and then exits from the distal paracentesis

**Fig. 3:**
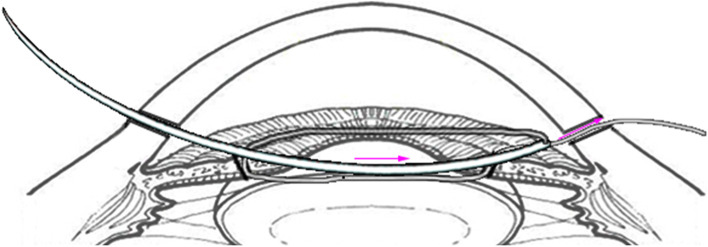
After the needle passes through two iris leaflets, its tail remains in the anterior chamber and then heads straight for the proximal paracentesis

**Fig. 4:**
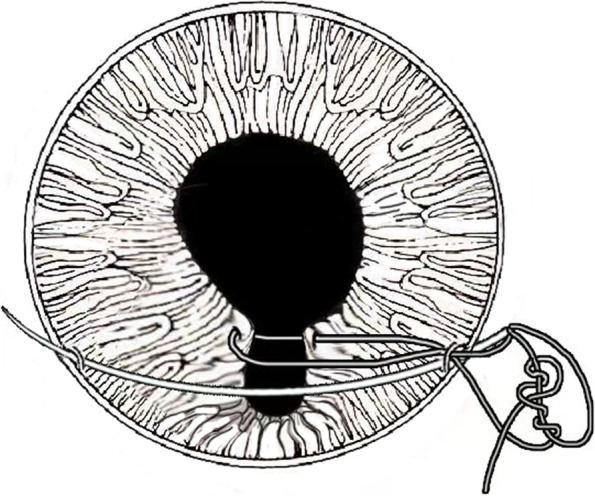
Pull the suture outside the proximal paracentesis to create a loop, wrapthree throws, pass the end of the suture through the second loop to prevent the knot loosening

**Fig. 5:**
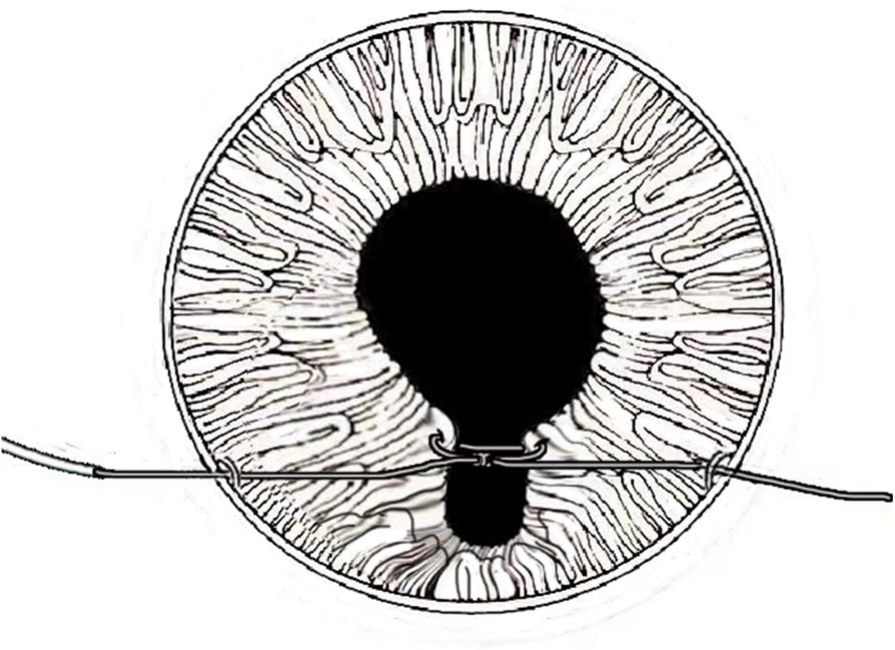
Slide the loops into anterior chamber, pull two free ends of suture inopposite directions equably and synchronously

**Fig. 6:**
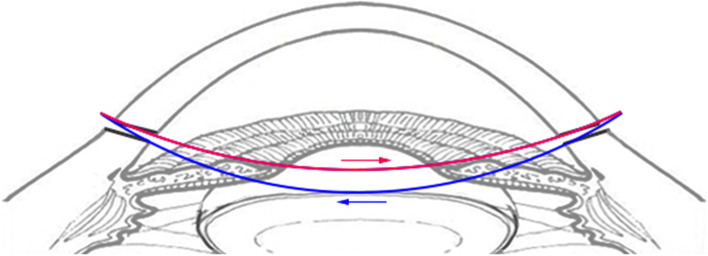
The threads in this surgery run like a crescent moon and the first letter of word crescent “C” is exactly like a new moon, so is called as C-pupilloplasty for short

**Fig. 7:**
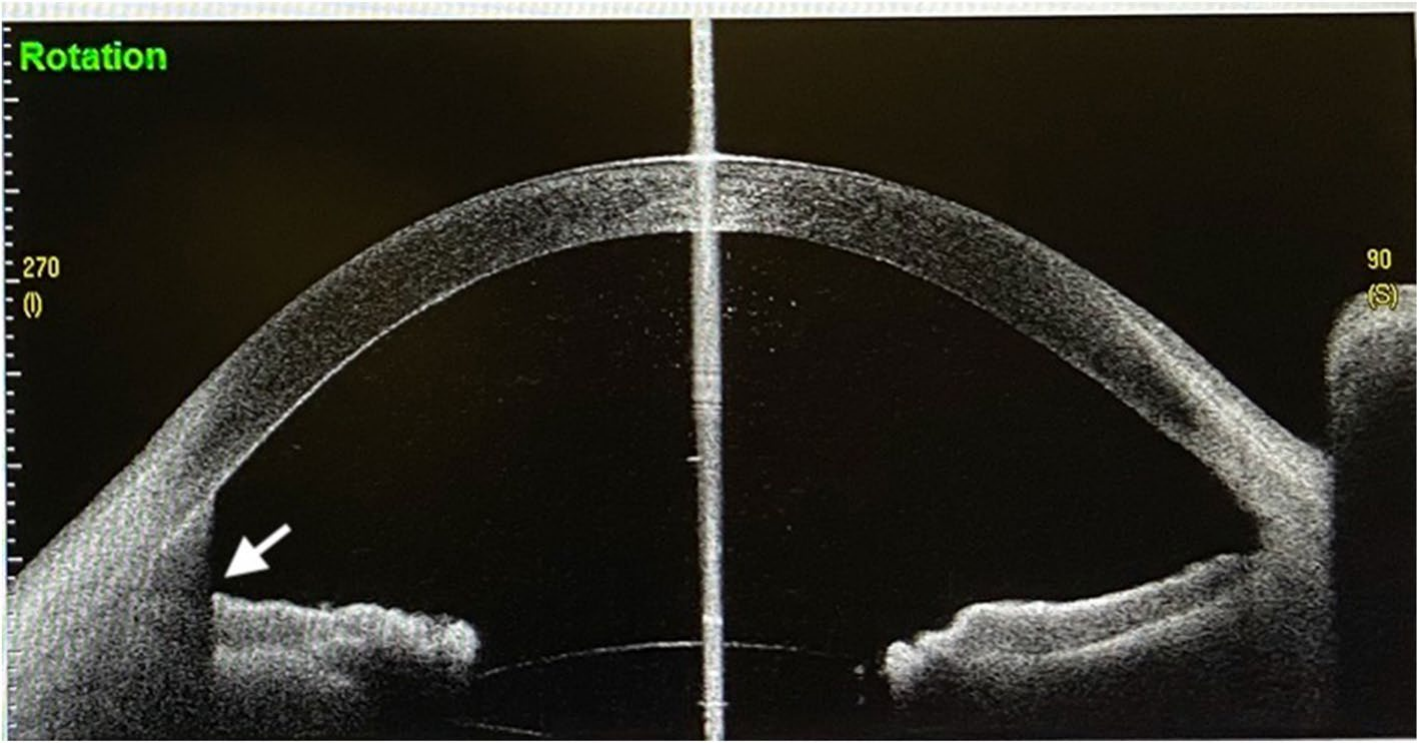
Vertical meridian scan with AS-OCT showed hyphema(arrow) of inferior anterior chamber 2 days after phacoemulsification, IOL implantation and pupilloplasty

All patients underwent a comprehensive evaluation including medical history, age, ocular and comorbid illnesses, range of iris coloboma or defect, size of pupil, complications, visual acuity, endothelial cell density, and preoperative symptoms such as glare, halos, photophobia, etc. The mean follow-up duration was 8.76 ± 3.58 months (ranging from 2 to 14 months). The C-pupilloplasty was performed with a single-pass two-throw technique in an attempt to make the maneuver easy and invasive. The PC-9 needle with a chord length of 13.5 mm was used (Alcon), which was tied toa10-0 polypropylene double-armed and 20-cm-long thread. Two 2.2 mm self-sealing paracentesis were first made on opposite corneal limbus positions perpendicular to the iris defect. Firstly, a viscoelastic agent was injected into the anterior chamber (AC), and the adhesions between iris and the cornea or lens were separated if needed. Next, a 27-gauge bent aspirating cannula with smooth blunt tip and end opening was introduced slowly from distal paracentesis into the AC, and exited the proximal one, to dock the tip of a PC-9 needle. This could prevent the needle from creating a separate tunnel near the paracentesis site. The docked needle tip entered the AC along with the cannula and was unreeved from the lumen. Once passing through the proximal and distal edges of the iris defect, the needle tip, which was again docked into the cannula, was driven out through the distal paracentesis until its tail went through the distal iris defect edge. The tip of the needle was then held outside the AC and the tail was sent directly to the proximal paracentesis until it protruded for at least 2 mm. The line connecting two limbal paracenteses was usually about 2.0 mm away from the corneal center. At this step, the whole needle was like a bow with its ends through the paracentesis on each site. The suture loop for wrapping the throw was created by pulling the thread near the tail, three throws are wrapped, the free end of the suture was threaded through the loop to prevent the knot from loosening, and then the knot was slid into the AC and tied (Figs. 1, 2, 3, 4, 5, 6 and 7).The knot must be always visualized, and kept in the center of defect iris, and tied by gentle force in opposite directions equally and synchronously. If one stitch was not enough to close the remaining iris defect, more sutures can be applied in the same way. Once the knot was tied, a microscissors was inserted into the AC to cut the suture, leaving the tails of about 1.0 mm left. Finally, the viscoelastic agent was extracted by irrigating and aspirating with Simcoe cannulas.

In trauma-induced iris defect associated with iridodialysis, there may still be a wide gap between the repaired pupil and limbus after suturing (double pupils). An iridodialysis repair should be performed before pupilloplasty. A paralimbic scleral groove is made to catch the peripheral iris edge in the groove incision, the thread is tied, and the knot is buried by closing the incision.

### ECD analysis

The center of cornea was examined using corneal endotheliometer (Topcon SP000P, Japan), to standardize the measurements, all images were subsequently randomized and encoded by a single independent observer. Each patient was measured three times and take the median. All the ECD were measured at 3 months after surgery except the 21th patient was 2 months.

### Statistical methods

The data were analyzed using IBM SPSS Statistics for Windows, Version20.0 (IBM Corp, Armonk, NY, USA) and reported as mean ± SD。Statistical analysis of comparison was performed by Paired *t*-test.

## Results

Among 21 patients, trauma and coloboma were found in nine and twelve cases, respectively. All the clinical data were shown in Table 1. Almost all patients complained of visual distortion, glare, and photophobia depending on the deformity, location and size of the abnormal pupil. Twelve patients presented with oval shaped and nine with keyhole shape pupils. In 20/21 patients, the pupils were repaired to a round or near round shape with a diameter less than or equal to 3 mm. But only one was still 4.5 mm in diameter after surgery due to multiple lacerations at the iris sphincter. Only one patient (No.5) presented with secondary intraocular hypertension postoperatively that continued for 3 weeks, and a positive Tyndall sign was found by slit-lamp microscopy and was also seen in the inferior angle by AS-OCT scanning (TOMEYCASIA SS-100, Tomey, Nagaya, Japan) (Fig. 7). She had a history of injury to her right eye hit by a tennis ball 8 years ago and diagnosed with traumatic angle recession, hyphema and hypertension. Her residual blood clot in the anterior chamber was absorbed completely, high intraocular pressure lowered to normal and visual acuity increased followed by removing of one iris thread located at iris frill, anti-glaucoma eye-drops, intravenous infusion of hemostatic solution and anterior chamber irrigation with Ringer solution. The preoperative discomforts related to irregular and dilated pupils such as dizziness, halo, glare and photophobia, disappeared rapidly after surgery in almost all patients. Even in one patient with a 4.5 mm pupil, the photophobia had become mild but negligible. The BCVA was increased from preoperative FC-0.1 to postoperative 0.3–0.8. The numbers of improved vision was 5.52 ± 1.27. The mean postoperative endothelial cell density was 2222.48 ± 458.08/mm^2^, which was only 3.25 ± 1.92 (%) slightly lower than the mean preoperative number of 2150.81 ± 449.06/mm^2^. No obvious inflammation or secondary cataract was seen during a median follow-up of 8.76 ± 3.58 months (ranging from 2 to 14 months). All patients were satisfied with the resolution of their symptoms and the improvement in pupil appearance (Table 1).

**Table 1:** The Characteristics of patients included in the study

	1	2	3	4	5	6	7	8	9	10	11	12	13	14	15	16	17	18	19	20	21
sex	M	F	M	M	F	F	M	M	M	M	M	F	M	M	F	F	M	M	M	M	M
age	26	48	18	21	48	30	61	27	57	56	41	20	76	41	42	39	69	33	52	70	96
eye	OD	OS	OS	OS	OD	OD	OD	OS	OS	OD	OS	OD	OS	OS	OD	OS	OS	OD	OS	OD	OS
Diagnosis																					
Traumatic	√	√	√		√		√		√	√		√		√			√		√		
Iatrogenic						√							√		√			√		√	√
Coloboma				√				√			√					√					
Follow-up(months)	4	12	7	9	12	12	7	3	9	7	14	13	11	11	6	4	7	13	11	10	2
Glare pre-op	mild	no	no	no	severe	mild	no	no	mild	moderate	mild	mild	no	mild	mild	no	no	moderate	severe	no	mild
Post-op	no	no	no	no	mild	no	no	no	no	no	no	no	no	no	no	no	no	no	no	no	no
Photophobia pre-op	severe	mild	no	no	severe	mild	mild	mild	mild	no	no	moderate	no	mild	mild	no	no	mild	severe	no	mild
Post-op	no	no	no	no	mild	no	no	no	no	no	no	no	no	no	no	no	no	no	mild	no	
Distorsion pre-op	mild	mild	mild	no	mild	mild	no	no	severe	mild	no	mild	no	no	no	severe	mild	no	mild	no	mild
Post-op	no	no	no	no	no	no	no	no	no	no	no	no	no	no	no	no	no	no	no	no	no
Pupil shape pre-op	keyhole	oval	oval	oval	oval	keyhole	keyhole	oval	round	oval	oval	round	round	oval	oval	keyhole	keyhole	oval	keyhole	oval	round
Post-op	round	oval	round	round	round	round	round	round	round	round	round	oval	round	oval	round	round	round	round	round	round	round
Vision pre-op	FC	0.1	0.08	0.06	0.1	0.1	0.05	0.06	0.1	FC	0.1	FC	0.08	0.1	0.2	0.08	0.04	0.1	0.1	0.1	0.1
Post-op	0.7	0.8	0.6	0.5	0.7	0.6	0.5	0.7	0.8	0.7	0.6	0.4	0.7	0.6	0.5	0.4	0.8	0.7	0.7	0.6	0.7
Numbers of improved vision	6.8	7	5.2	4.4	6	5	4.5	6.4	7	6.8	5	3.8	6.2	5	3	3.2	7.6	6	6	5	6
Pupil size(mm) pre-op	7*9	7*6	7*7	6*9	6*9	6*9	7*9	5*8	7*7	8.5*8	7*8	8*8	7.5*7.5	6*7	7*7	6*8.5	5.5*9	6*7.5	4*7	6*7.5	8*8
Post-op	4*4	3.5*3.5	3.5*3.5	5*.5	5*5	4.5*4.5	3*3	3*3	4*4	3.5*3.5	3.5*3.5	3*4	3.5*3.5	3*4	3.5*3.5	3.5*3.5	3.5*3.5	3.5*3.5	3.5*3.5	3.5*3.5	3.5*3.5
ECD pre-op	3003	2474	2783	2988	1987	2084	1782	2006	3003	1898	2338	1640	2003	2681	1840	1663	2103	2640	2050	1726	1980
Post-op	2894	2444	2559	2891	1928	2001	1705	1893	2994	1864	2229	1576	1994	2634	1799	1577	1997	2591	1989	1709	1898

## Discussion

Laceration or defect of the iris may result in anterior synechiae, mydriasis, endothelial rubbing or other complications. Pupilloplasty is indicated to reduce the size of the deformed pupil with a diameter of more than 5mm caused by congenital diseases or trauma. This technique helps to minimize aberration, diffraction, glare and photophobia, to enhance the pinhole effect, to expand the depth of field, to restore the normal optical axis as well as to improve the cosmetic appearance. The iris suturing technique was first introduced by McCannel in 1976, in which three minor limbal paracenteses were used [4] .In this procedure, the iris is stretched as it is pulled toward the limbal paracentesis tract when the suture thread is cut. In 1994, Siepser et al [1, 13] reported an improved method based on the McCannel technique with only two paracenteses and slip-knot tiring, which could reduce iatrogenic astigmatism and the risk of iris tearing. But the slip-knot may be loosened if a second knot is not tied and locked tighter, which increases the possibility of injuring the endothelium and infection due to multiple entry into the anterior chamber with the surgical instruments. Yao [2] modified the pupilloplasty technique so that only one limbal paracentesis was employed to treat the iris defect within the 120° range in 2017.The technique can reduce the incidence of corneal damage, but a hook inside the anterior chamber and a double knot were required, which may increase mechanical damage to the endothelium. But if two or more sutures are needed, it is very difficult to pass through the previous limbal passage. Most previous approaches had been performed with a single straight needle and thread.

Frequent entry of surgical instruments into the anterior chamber can cause certain problems such as endothelial damage. In our technique, a blunt cannula is inserted behind the proximal iris defect to facilitate the passage of the needle through the flimsy iris edge to dock into the cannula. Similarly, the needle tip passes the distal margin of the iris defect from behind and dock into the cannula. It is important to identify and manage the iris elasticity and to release the anterior or posterior iris synechia before suturing, otherwise iris tear tends to occur. There is a risk that the iris maybe broken or the knot may not be tightened firmly with this technique. If corneal topography or keratometry shows corneal astigmatism before surgery, limbal relaxing paracenteses can be made as close as possible near the axis of the steepest refractive power without affecting iris suturing and knot tying in a closed chamber. Because the tail of the needle is blunt and round, when it exits from the proximal limbal tunnel, it will not penetrate the matrix outside the tunnel. However, in order to ensure that the tail of the needle does not deviate from the tunnel, a cannula can be inserted from the proximal paracentesis into its inner opening, and the tail of the needle enters cannulae a little distance, and then move them together outside the tunnel synchronously. Inserting the tail of the needle into the cannule does not generate shearing force so the sutures are less likely to break.

To the best of our knowledge, this is the first study to use 10/0 polypropylene double armed thread attached to the Alcon PC-9 needle in pupilloplasty. Compared to other methods [3, 14–16]. Some surgical instruments such as Sinskey hook, dialer, microforceps or straight needles are not required in our technique. A long and curved needle can effectively avoid the stabbing on the lens and unwanted traction on the iris tissue during the passage of the needle because its sharp tip is always pointed up or side ways. During the withdrawal of the blunt cannula from the Proximal paracentesis, be careful not to allow the tip of needle to be released in the anterior chamber from the distal paracentesis since it could trigger the endothelium or even the lens loss. A three-throw is enough to form a tight knot to secure bilateral defect pupils and makes cheese-wiring of the iris less likely by the suture since threads are not needed on both sides. Taking out the suture without a hook or dialer inside the anterior chamber will also reduce the chance of damaging the iris and corneal endothelium. More sutures can be placed in the same wayif the diameter of pupil is still large or the iris cleft is too wide. It should be noted that the needle could not pass through the lesser arterial circle of iris, which is characterized by circulus iridis on the iris surface about 1.5 mm from the pupillary margin and presents annular ridges in the form of gear wheel. However, in some cases with iris coloboma or a traumatic dilated pupil, the feature of iris collarette is not obvious. In this case, it is advisable to suture the defected iris at least 3 mm away from the pupillary margin. Supposing that aqueous humor inflammation or even hyphema occurs shortly after surgery, a lesser arterial circle of the iris should be firstly suspected to be damaged during needle passing, and the sutures near iris collarette should be removed as early as possible.

The BCVA was increased from preoperative FC-0.1 to postoperative 0.3-0.8. The numbers of improved vision was 5.52±1.27, which showed remarkable progress in patients’ visual acuity. The mean postoperative ECD was 2222.48±458.08/mm^2^, while the preoperative was 2150.81±449.06/mm^2^, The ECD loss was only 3.25±1.92(%). The superiority of this technique was fully illustrated.

The main limitation of this technique is the limited chord length of PC-9 needle. The13.5-mm chord length is suitable for pupilloplasty in almost all normal-sized cornea except the cornea greater than 13.5 mm in diameter such as macrocornea, keratoglobus and anterior megalophthalmos [17]. On this occasion, an Alcon AUM-5 17-mm-straight needle with a double-armed 10/0 polypropylene suture can be used. We recommend that the straight needle to be bent into a mild arc to facilitate passing through the defected iris intracameral. Another limitation is the larger iris loss where the residual iris is not elastic enough to close the defect by suture. In this case, artificial iris implantation is required [18].

## Conclusion

The C-pupilloplasty is a simplified, easily-to-learn and cost-effective technique for iris coloboma and traumatic defect. A 13.5-mm chord length needle will facilitate the passage from the limbal paracentesis and make the operation easy, which can minimize the incidence of iatrogenic cataracts. No special surgical instruments are required for the technique, and the whole procedure takes only 10-15 minutes. The C-pupilloplasty provides a safer, more convenient and reasonable option for pupillary reconstruction.

## Data Availability

The data that support the findings of this study are available on request from the corresponding author.

## Abbreviations

FC: Finger counting
BCVA: Best corrected visual acuity
ECD: Endothelial cell density
